# A Comparison of KL-6 and Clara Cell Protein as Markers for Predicting Bronchopulmonary Dysplasia in Preterm Infants

**DOI:** 10.1155/2014/736536

**Published:** 2014-08-27

**Authors:** Keyi Wang, Xianmei Huang, Hui Lu, Zhiqun Zhang

**Affiliations:** ^1^Department of Central Laboratory, Hangzhou First People's Hospital, No. 261 Huansha Road, Hangzhou, Zhejiang 310002, China; ^2^Department of Pediatrics, Hangzhou First People's Hospital, No. 261 Huansha Road, Hangzhou, Zhejiang 310002, China; ^3^Division of Neonatology, Department of Pediatrics, Hangzhou First People's Hospital, No. 261 Huansha Road, Hangzhou, Zhejiang 310002, China

## Abstract

*Objectives*. To evaluate the predictive characteristics of KL-6 and CC16 for bronchopulmonary dysplasia (BPD) in preterm infants, either independently or in combination.* Study Design*. This prospective cohort study was performed from 2011 to 2013 with preterm neonates of gestational age ≤32 weeks and birth weight ≤1500 g. Serum KL-6 and CC16 levels were determined 7 and 14 days after birth.* Results*. Seventy-three preterm infants were studied. BPD was identified in 24 of these infants. After adjusting for potential confounders, serum KL-6 concentrations were found to be elevated in BPD infants at both time points relative to non-BPD infants, while serum CC16 concentrations were lower at 14 days. At both 7 d and 14 d of life the predictive power of KL-6 levels exceeded that of CC16 (area under receiver operating characteristic curve: at 7 d, 0.91 cf. 0.73, *P* = 0.02; at 14 d, 0.95 cf. 0.85, *P* = 0.05). The combination of these markers enhanced the sensitivity further.* Conclusions*. Serum KL-6 levels higher than 79.26 ng/mL at 14 days postpartum in preterm infants predict the occurrence of BPD. CC16 was less predictive than KL-6 at this time point, but KL-6 and CC16 together enhanced the prediction.

## 1. Introduction

Bronchopulmonary dysplasia (BPD) is a syndrome of respiratory distress caused by chronic lung parenchymal injury, occurring primarily in preterm infants [1]. The hallmark of BPD is a requirement for either oxygen therapy or positive pressure ventilation for 4 or more weeks after birth [2, 3]. However, for extremely premature infants born at 25 or 26 weeks' gestation, dependence on oxygen could mean lung immaturity rather than lung injury, and therefore more direct evidence of lung parenchymal injury upon which to rest a diagnosis of BPD is needed [4]. Chest radiographs do not necessarily indicate the extent of lung damage, and their interpretation is subjective [5, 6]. Ideally, a means of obtaining a definitive diagnosis of BPD should rely on simple clinical observations, or specific biological markers that grade severity, is generalizable, and is able to predict late pulmonary morbidity and neurodevelopmental outcomes. Therefore, a specific and objective marker that accurately reflects the severity of lung injury is very much needed.

Circulating KL-6 levels are markedly higher in patients with interstitial lung diseases and interstitial pneumonia, diseases which are characterized by type-II alveolar hyperplasia and fibrosis. Similar pathological changes in the lung are predominant in BPD [7–10]. Furthermore, infants with BPD have significantly higher plasma KL-6 levels compared with those without BPD [11–15]. Ogihara et al. [14] reported that plasma KL-6 levels at 2 weeks after birth were an excellent predictor of BPD (sensitivity 83%, positive predictive value 80%). However, the KL-6 values for predicting BPD reported in that study did not concur with those of another recent study using the same KL-6 kit and method [15].

The putative role of Clara cell protein (CC16), another peripheral blood biomarker originating from nonciliated Clara cells, for predicting BPD was explored in a recent study [16]. Low cord blood concentrations of CC16 were reported to independently predict the development of BPD in preterm infants. In contrast, Sarafidis et al. [17] found that CC16 concentrations at 2 h, 72 h, and 14 d were significantly higher in ventilated infants who developed BPD than in those who did not.

A systematic review suggested that plasma KL-6 and CC16 levels could be useful as early markers for predicting BPD in preterm infants [18]. It has also been suggested that a combination of biomarkers could be more sensitive than only one [19, 20]. The present prospective study was performed to determine a reference range for plasma KL-6 and CC16 for predicting BPD and to evaluate the predictive efficacy of combinations of KL-6 and CC16.

## 2. Subjects and Methods

The ethics committee of our hospital approved the study protocol. Investigations were performed only after parents of the study subjects provided informed consent.

### 2.1. Study Population

All eligible preterm infants between June 2011 and July 2013 were prospectively enrolled in this study, specifically neonates with birth weight ≤1500 g and gestational age ≤32 weeks, and admitted to the neonatal intensive care unit of Hangzhou First People's Hospital. Exclusion criteria included those with congenital heart disease, multiple malformations, or a documented chromosomal abnormality. Also excluded from the final analysis were infants who died of nonrespiratory causes or were discharged before reaching a postconceptional age of 36 weeks, or for whom blood samples were not obtained at both 7 and 14 d postpartum.

The following demographic and perinatal characteristics were recorded: gender, birth weight, gestational age, pregnancy-induced hypertension, diabetes, premature rupture of membranes >24 h, prenatal steroid administration, mode of delivery, Apgar score, and cord blood gas details. Also recorded were the following pulmonary data: surfactant administration, days of mechanical ventilation, days of supplemental oxygen, and need for supplemental oxygen or mechanical ventilation at 28 d and 36 weeks' postmenstrual age.

BPD was defined in accordance with guidelines of the National Institute of Child Health and Human Development/National Heart, Lung, and Blood Institute Workshop (National Institutes of Health consensus definition) for infants born at gestational age <32 weeks, that is, treatment with >21% oxygen for at least 28 d [21]. At 36 weeks' postconceptional age, the infants were classified as mild, moderate, or severe BPD based on the required fraction of inspired oxygen (FiO_2_): mild BPD, none; moderate, 21% to 30%; and severe, >30% or positive pressure assistance [21].

### 2.2. Pulmonary and Supportive Care

These preterm infants received respiratory support as per the European Consensus Guidelines on the Management of Neonatal Respiratory Distress Syndrome in Preterm Infants—2010 Update [22]. Exogenous surfactant (Curosurf, 100–200 mg*·*kg^−1^ *·*dose^−1^) was given within the first 6 h after birth and two further doses. Prophylactic surfactant administration in the delivery room was not administered. After extubation, infants were administered nasal continuous positive airway pressure. Mechanical ventilation was used to support neonates with respiratory failure, as this improves survival. Crossover treatment, from conventional ventilation to high-frequency oscillatory ventilation, was applied to neonates with refractory respiratory failure. In general, supportive treatment during the study was at the discretion of the attending neonatologist, in accordance with the unit protocols.

### 2.3. Blood Sampling and Plasma KL-6 and CC16 Measurements

We obtained heparinized blood samples from the infants by venipuncture at postpartum days 7 and 14 during hospitalization (mostly, we salvaged the residual blood that was obtained for routine examinations). The blood samples were immediately centrifuged at 3000 ×g for 10 min at 4°C to obtain plasma and then stored at −80°C. The KL-6 and CC16 levels in plasma were measured using a quantitative colorimetric sandwich enzyme-linked immunosorbent assay kit (Blue Gene, China) in accordance with the manufacturer's instructions. Each sample was run in duplicate and the mean concentration was calculated. The sensitivities as per the kit for KL-6 and CC16 were 0.1 ng/mL and 1.0 pg/mL, respectively.

### 2.4. Statistical Analyses

Numerical data are expressed as median with range, as not all of the parameters studied followed a normal distribution (Kolmogorov-Smirnov test). Differences in numeric variables were assessed using Mann-Whitney, Friedman's, or Kruskal-Wallis nonparametric two-tailed tests, with post hoc analysis (Dunn's multiple comparison tests) when indicated. Fisher's exact test was used for the categorical variables. Multiple linear regressions were used to assess the influence of demographic and perinatal characteristics on CC16 and KL-6 levels at different time points. To validate the usefulness of KL-6 and CC16 in predicting BPD, receivers operating characteristic (ROC) curves were calculated at different time points and cut-off levels were determined when a significant result was obtained. Values of *P* < 0.05 were considered significant. All statistical analyses were performed using SAS 9.2 software (SAS Institute, Cary, NC, USA).

## 3. Results

### 3.1. Infant Characteristics

**Figure 1:**
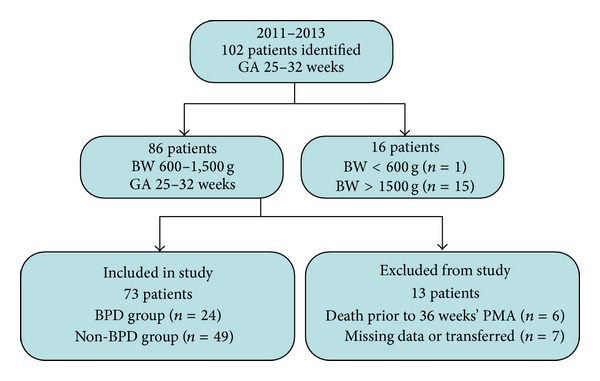
Inclusion and exclusion of patients.

During the study period, 86 infants met the inclusion criteria. Among these preterm infants, 6 died and 7 were transferred or had missing data before reaching 36 weeks' postconceptional age. Finally, 73 infants were included in the study; 24 infants (gestational age: 29.2 ± 1.7) had BPD and 49 infants did not (Table 1, Figure 1). BPD was mild in 13 infants and moderate or severe in 11. Compared with infants without BPD, those with BPD had significantly lower mean birth weight (*P* < 0.0001) and a higher incidence of surfactant use (*P* = 0.03), mechanical ventilation ≥1 week (*P* = 0.003), and sepsis (*P* = 0.003), as well as longer hospitalization (*P* = 0.0001).

### 3.2. Levels of CC16 and KL-6

**Figure 2:**
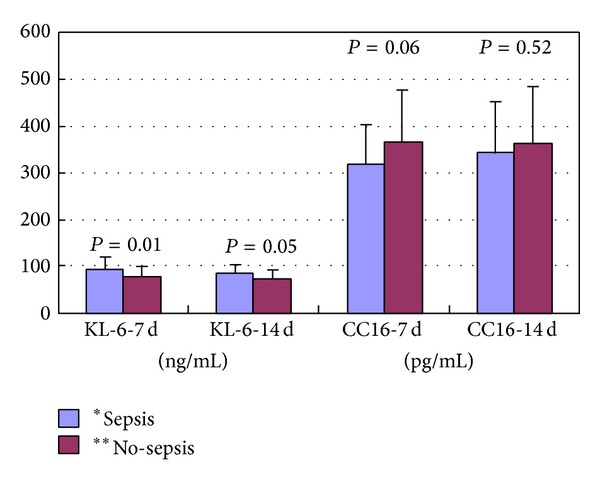
KL-6 and CC16 levels in infants with sepsis and those without sepsis at postpartum days 7 and 14. *Sepsis: sepsis occurred within 15 days after birth. **No-sepsis: no sepsis occurred within 15 days after birth.

**Figure 3:**
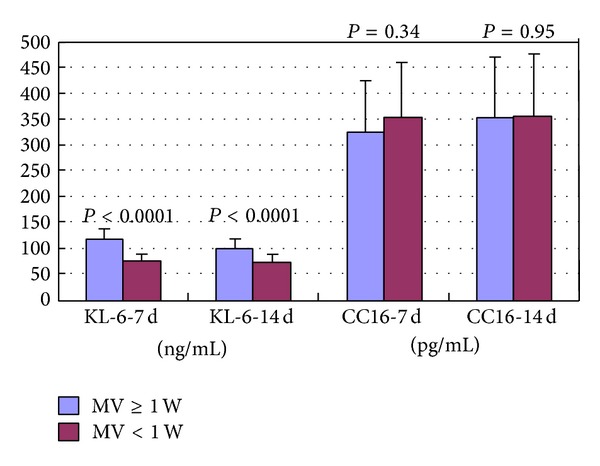
KL-6 and CC16 levels in infants with mechanical ventilation ≥1 week and those with mechanical ventilation <1 week at postpartum days 7 and 14. MV: mechanical ventilation.

Plasma KL-6 levels were not significantly correlated with gestational age (at 7 days: *r* = −0.202, *P* = 0.085; at 14 days: *r* = −0.084, *P* = 0.478) (Table 2) but showed a significant correlation with birth weight (at 7 days: *r* = −0.452, *P* < 0.001; at 14 days: *r* = −0.424, *P* < 0.001) (Table 2), sepsis (at 7 days: *P* = 0.01; at 14 days: *P* = 0.05) (Figure 2), and mechanical ventilation ≥1 week (at 7 days: *P* < 0.0001; at 14 days: *P* < 0.0001) (Figure 3). Plasma CC16 levels were not significantly associated with gestational age (at 7 days: *r* = 0.104, *P* = 0.379; at 14 days: *r* = 0.072, *P* = 0.542) (Table 2), sepsis (at 7 days: *P* = 0.06; at 14 days: *P* = 0.52) (Figure 2), or mechanical ventilation ≥1 week (at 7 days: *P* = 0.34; at 14 days: *P* = 0.95) (Figure 3) but showed a significant correlation with birth weight (at 7 days: *r* = 0.286, *P* = 0.014; at 14 days: *r* = 0.245, *P* = 0.036) (Table 2).

**Figure 4:**
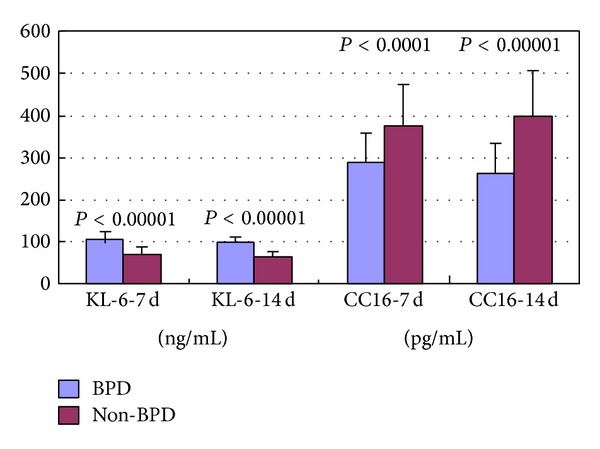
KL-6 and CC16 levels in infants with BPD and those without BPD at postpartum days 7 and 14.

At postpartum days 7 and 14, the KL-6 levels in infants with BPD (107.65 ± 19.1 ng/mL and 98.67 ± 13.7 ng/mL, resp.) were significantly higher than these levels in infants without BPD (71.84 ± 13.8 ng/mL and 66.58 ± 12.7 ng/mL; *P* < 0.0001, both). On the other hand, at postpartum days 7 and 14, the CC16 levels in infants with BPD (288.96 ± 68.9 pg/mL and 262.31 ± 69.2 pg/mL, resp.) were significantly lower than these levels in infants without BPD (373.98 ± 101.2 pg/mL and 399.82 ± 105.0 pg/mL; *P* < 0.0001, both; Figure 4).

Multivariable logistic regression analysis with BPD as the dependent variable revealed that KL-6 levels at 7 d (odds ratio [OR]: 1.16, 95% CI: 1.07–1.26; *P* < 0.001), KL-6 levels at 14 d (OR: 1.76, 95% CI: 1.05–3.01; *P* < 0.05), and CC16 levels at 14 d (OR: 0.82, 95% CI: 0.76–0.90; *P* < 0.001) were independently correlated (Table 3).

When BPD subgroups, mild and moderate/severe, were analyzed there was no difference in mean gestational age between the mild BPD and moderate/severe BPD subgroups (*P* = 0.12). However, compared with the mild subgroup, the KL-6 levels of the moderate/severe subgroup were significantly higher at postpartum days 7 (*P* < 0.0001) and 14 (*P* = 0.04). The CC16 levels of the subgroups were similar at both time points (*P* = 0.93 and *P* = 0.14, resp.; Table 4).

### 3.3. Prediction of BPD by KL-6 and CC16

**Figure 5:**
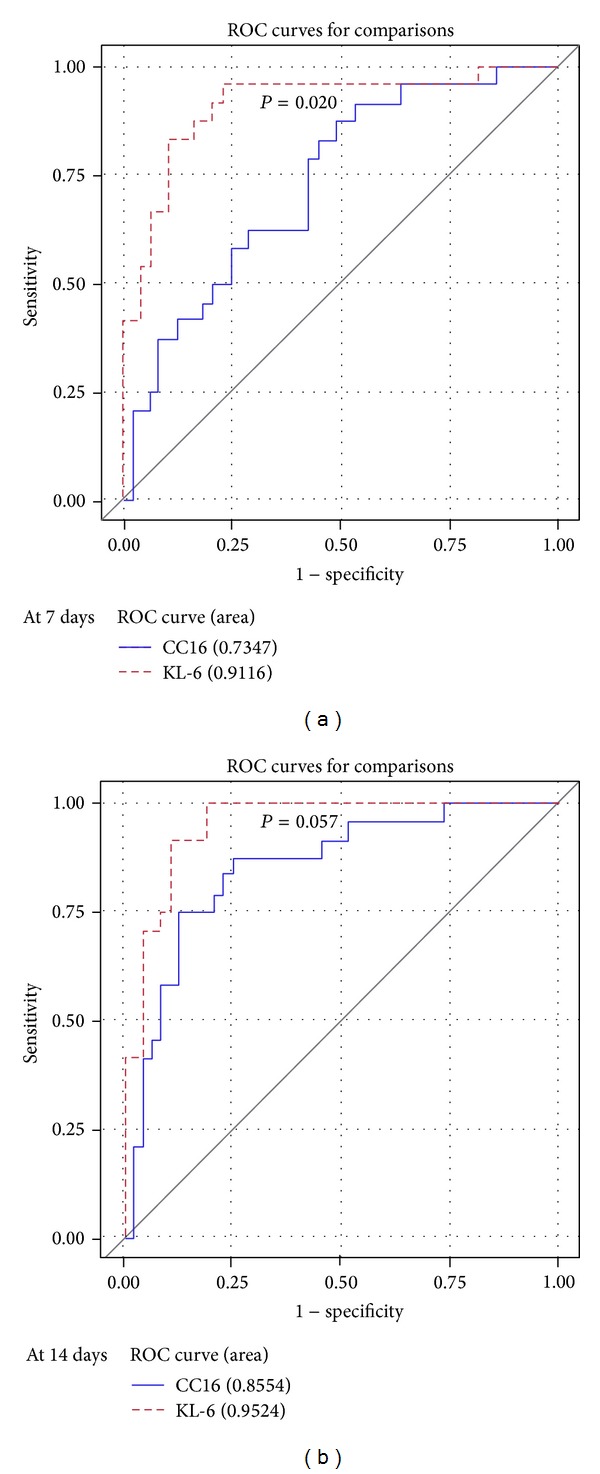
Comparison of receiver operating characteristic curves for plasma KL-6 and CC16 levels at day 7 and day 14 of life.

Using cut-off values selected with ROC curves, we compared the predictive ability of KL-6 with that of CC16 for the development of BPD. The KL-6 levels at postpartum days 7 and 14 had better predictive power than did CC16 levels (Figure 5). The best single predictor was KL-6 levels at postpartum day 14 (area under the ROC curve [AUC] = 0.952; *P* < 0.001). When KL-6 and CC16 combination was considered, KL-6 and CC16 levels on day 14 had the best predictive characteristics (AUC = 0.974, range 0.82–1.03; *P* < 0.001; Table 5).

## 4. Discussion

In this prospective cohort study, we found that serum KL-6 was elevated and CC16 was decreased at postpartum days 7 and 14 in preterm infants who subsequently developed BPD. Moreover, the concentrations of serum KL-6 seemed to be closely related with the severity of BPD. On the contrary, serum CC16 seemed to be having no relation with the severity of BPD. It was also observed that serum KL-6 concentrations entail important predictive value for subsequent BPD development at postpartum days 7 and 14.

We found in the present study that plasma KL-6 and CC16 levels were not influenced by gestational age, but they correlated with mechanical ventilation ≥1 week and sepsis. These results indicate that changes in plasma KL-6 and CC16 levels are associated with lung injury and infection. After adjusting for confounding risk factors, KL-6 and CC16 levels still correlated significantly with BPD incidence. When infants with BPD were further stratified by BPD severity, there was no difference between the mild and moderate/severe subgroups with regard to gestational age. However, infants with moderate/severe BPD had higher KL-6 levels (at postpartum days 7 and 14), while there were no significant differences in CC16 levels. This suggests that changes in plasma KL-6 levels were precipitated by the infants' lung injury. These changes suggest that the lung of the preterm infant is vulnerable to injury. Higher serum KL-6 levels were associated with worse pulmonary outcome (i.e., BPD) and were possibly related to worse long-term prognosis [10]. The lower plasma CC16 concentrations did not correlate with disease severity in this study. This is in contrast to a previous report of a positive correlation between lower CC16 in cord blood and disease severity [16]. The discord in these findings may be due to some extent by the timing of blood specimens. Low cord blood levels of CC16 in preterm infants may reflect prenatal lung injury. Ogihara et al. [14] suggested that prenatal lung injury has a priming effect, increasing the susceptibility of fetal lungs to subsequent toxic agents. Loughran-Fowlds et al. [23] also found that infants who developed BPD had persistently lower serum CC16 levels at 12 hours compared with those who did not develop BPD. However, Sarafidis et al. [17] found significantly higher CC16 concentrations at 14 days in ventilated infants who developed BPD than in nonventilated infants who did not develop BPD. These inconsistent results regarding CC16 levels may be attributed to unstable plasma concentrations. A transient increase in serum CC16 levels following acute exposures to certain pulmonary irritants had been reported [24]. As a lung protective mechanism, CC16 may be consumed when the inflammation persists leading to its lower levels [24].

It has been reported previously that BPD preterm neonates have higher plasma KL-6 levels compared with preterm neonates without BPD [8, 11, 14], but data on the predictive characteristics of KL-6 are scarce. Ogihara et al. [14] in their study of 42 infants at <28 weeks of gestational age reported that serum KL-6 levels ≥232 U/mL at postpartum 14 days had an AUC of 0.8995, a sensitivity of 84%, and a specificity of 82% for predicting BPD. The results of the present study showed KL-6 levels of 79.26 ng/mL at day 14 with a sensitivity of 91.7% and a specificity of 89.8% for subsequent BPD. The cut-off plasma values for KL-6 of these two studies cannot be compared because different kits were used. However, the similarities in the observations suggest that plasma KL-6 concentrations are relatively stable and significantly increase only under conditions associated with lung injury.

The present study is the first to evaluate the potential role of combined KL-6 and CC16 for predicting BPD in preterm infants. When the ROC curves generated by the KL-6 and CC16 data were analyzed as markers for subsequent BPD in preterm infants, it was found that plasma KL-6 levels were the better predictor, at both 7 and 14 days postpartum. Analysis of the combined KL-6 and CC16 data showed that together they predicted BPD better than either alone. Biomarkers predicting subsequent BPD allow the clinician to plan for early interventions to prevent the disease. In an attempt to minimize lung injury, the clinician can use lung-protective ventilatory strategies [25] or certain pharmacological agents [26].

Our results in the present study may be considered limited by the relatively small sample size, which could have hidden small but clinically relevant differences in some of the outcome parameters. In addition, we used the residual blood after routine examinations for the estimation of biomarkers and some specimens might have undergone hemolysis, influencing the test results. Finally, the study did not perform long-term follow-ups for pulmonary and neurodevelopmental outcomes in these neonates.

In conclusion, in the present study we found that serum KL-6 levels at 14 days postpartum in preterm infants could predict the subsequent occurrence of BPD, and a combination of KL-6 and CC16 levels further enhanced the predictive value. The predictive abilities of these markers appear to be clinically useful and may allow early therapeutic intervention. However, the clinical significance of using both KL-6 and CC16 levels for monitoring BPD in preterm neonates should be validated in larger studies.

## Figures and Tables

**Table 1:** Comparison of demographic and clinical characteristics between infants with and without BPD*.

	BPD	Without BPD	*P* value
Number of patients	24	49	
Birth weight, g	1124.6 ± 216.8	1311.8 ± 115.6	<0.0001

Gestational age, weeks	29.2 ± 1.7	31.2 ± 1.0	0.07
Male	15 (62.5%)	22 (44.9%)	0.16

Apgar score at 1 min	5.5 [3.5–7]	7 [5–7]	0.14
Apgar score at 5 min	6 [5–8]	7 [7-8]	0.10

Apgar score at 10 min	7 [7-8]	8 [8-8]	0.07
Vaginal delivery	7 (29.2%)	9 (18.4%)	0.3

Prenatal steroids	23 (95.8%)	44 (89.8%)	0.39
Maternal pregnancy-induced hypertension	7 (29.2%)	18 (36.7%)	0.52

Maternal diabetes	2 (8.3%)	1 (2.0%)	0.24
Maternal infection	2 (8.3%)	4 (8.2%)	0.98

Premature rupture of membranes	11 (45.8%)	13 (26.5%)	0.1
Cord blood pH	7.30 ± 0.08	7.30 ± 0.08	1.0

Ureaplasma urealyticum positive in urine	3 (12.5%)	12 (13.3%)	0.56
Surfactant use	23 (95.8%)	31 (34.4%)	0.03

Mechanical ventilation ≥1 week	11 (45.8%)	4 (8.2%)	0.003
Pneumonia	4 (16.7%)	3 (6.1%)	0.17

Sepsis	18 (75.0%)	22 (44.9%)	0.02
Sepsis occurred within 15 days after birth	16 (66.7%)	14 (28.6%)	0.003
Sepsis occurred after 15 days after birth	2 (8.3%)	8 (16.3%)	0.36
Patent ductus arteriosus	6 (25.0%)	4 (8.2%)	0.06

Length of stay, d	49.9 ± 11.2	40.3 ± 6.6	0.0001
Patients discharged alive	20 (83.3%)	49 (100.0%)	0.04

*Values are expressed as median [range] or number of individuals (percentage).

**Table 2:** Associations of serum KL-6 and CC16 (dependent variable) with selected clinical variables (independent variable) by multiple linear regression analysis.

	Age∗		*r*-value	*P* value
KL-6	7 d	Gestational age	−0.202	0.085
		Birth weight	−0.452	<0.001
	14 d	Gestational age	−0.084	0.478
		Birth weight	−0.424	<0.001

CC16	7 d	Gestational age	0.104	0.379
		Birth weight	0.286	0.014
	14 d	Gestational age	0.072	0.542
		Birth weight	0.245	0.036

*Postpartum.

**Table 3:** Association between plasma KL-6 and CC16 levels with BPD after adjusting for birth weight, surfactant use, mechanical ventilation ≥1 week, and sepsis, as computed by logistic regression analysis.

	Age (d)∗	aOR	95% CI	*P* value
KL-6	7	1.16	1.07–1.26	0.0004
CC16		0.99	0.98–1.01	0.07
KL-6	14	1.76	1.05–3.01	0.04
CC16		0.82	0.76–0.90	0.0006

*Postpartum.

**Table 4:** Gestational age, birth weight, and serum KL-6 and CC16 levels of the mild and moderate/severe BPD subgroups.

	Age (d)∗	Mild BPD	Moderate/severe BPD	*P* value
Number of patients		13	11	

Gestational age, weeks		29.7 ± 1.7	28.6 ± 1.6	0.12

Birth weight, g		1188.4 ± 233.5	1049.1 ± 176.4	

KL-6 (ng/mL)	7	92.0 ± 15.3	126.2 ± 15.8	<0.0001
	14	92.7 ± 13.4	105.8 ± 16.5	0.04

CC16 (pg/mL)	7	290.6 ± 103.2	286.9 ± 71.3	0.93
	14	238.8 ± 87.5	289.9 ± 79.9	0.14

*Postpartum.

**Table 5:** KL-6 (ng/mL) and CC16 (pg/mL) cut-off levels for predicting BPD.

	Age (d)∗	Cut-off	Sensitivity (%)	Specificity (%)	Predictive value (%)		AUC
					Positive	Negative	
KL-6	7	≥89.347	83.3	89.8	80.0	91.7	0.911
	14	≥79.261	91.7	89.8	81.5	95.7	0.952

CC16	7	≤317.26	71.4	62.5	51.6	80.5	0.734
	14	≤358.35	75.5	87.5	67.7	90.3	0.855

KL-6 + CC16	7		100	96.7	100	100	0.932
	14		100	97.1	100	100	0.974

*Postpartum.
